# Long-term and realistic global change manipulations had low impact on diversity of soil biota in temperate heathland

**DOI:** 10.1038/srep41388

**Published:** 2017-01-25

**Authors:** Martin Holmstrup, Christian Damgaard, Inger K. Schmidt, Marie F. Arndal, Claus Beier, Teis N. Mikkelsen, Per Ambus, Klaus S. Larsen, Kim Pilegaard, Anders Michelsen, Louise C. Andresen, Merian Haugwitz, Lasse Bergmark, Anders Priemé, Andrey S. Zaitsev, Slavka Georgieva, Marie Dam, Mette Vestergård, Søren Christensen

**Affiliations:** 1 Department of Bioscience, Aarhus University, Vejlsøvej 25, 8600 Silkeborg, Denmark; 2 Aarhus Institute of Advanced Studies, Aarhus University, Høegh-Guldbergs Gade 6B, 8000 Aarhus C, Denmark; 3 Section for Forest, Nature and Biomass, Department of Geosciences and Natural Resource Management, University of Copenhagen, Rolighedsvej 23, 1958 Frederiksberg, Denmark; 4 Department of Environmental Engineering, Atmospheric Environment, Technical University of Denmark, 2800 Kgs, Lyngby, Denmark; 5 Section for Geography, Department of Geosciences and Natural Resource Management, University of Copenhagen, Øster Voldgade 10, 1350 Copenhagen, Denmark; 6 Terrestrial Ecology Section, Department of Biology, University of Copenhagen, Universitetsparken 15, 2100 Copenhagen Ø, Denmark; 7 Department of Earth Sciences, University of Gothenburg, P.O. Box 460, 405 30 Gothenburg, Sweden; 8 Section of Microbiology, Department of Biology, University of Copenhagen, Universitetsparken 15, 2100 Copenhagen Ø, Denmark; 9 Institute of Animal Ecology, Justus-Liebig-University, Heinrich-Buff Ring 26-32 (IFZ), 35392 Giessen, Germany; 10 A.N. Severtsov Institute of Ecology and Evolution, Russian Academy of Sciences, Leninsky prospekt, 33, Moscow 119071, Russian Federation; 11 Department of Zoology and Anthropology, Faculty of Biology, Sofia University, Dragan Tzankov 8, 1164 Sofia, Bulgaria

## Abstract

In a dry heathland ecosystem we manipulated temperature (warming), precipitation (drought) and atmospheric concentration of CO_2_ in a full-factorial experiment in order to investigate changes in below-ground biodiversity as a result of future climate change. We investigated the responses in community diversity of nematodes, enchytraeids, collembolans and oribatid mites at two and eight years of manipulations. We used a structural equation modelling (SEM) approach analyzing the three manipulations, soil moisture and temperature, and seven soil biological and chemical variables. The analysis revealed a persistent and positive effect of elevated CO_2_ on litter C:N ratio. After two years of treatment, the fungi to bacteria ratio was increased by warming, and the diversities within oribatid mites, collembolans and nematode groups were all affected by elevated CO_2_ mediated through increased litter C:N ratio. After eight years of treatment, however, the CO_2_-increased litter C:N ratio did not influence the diversity in any of the four fauna groups. The number of significant correlations between treatments, food source quality, and soil biota diversities was reduced from six to three after two and eight years, respectively. These results suggest a remarkable resilience within the soil biota against global climate change treatments in the long term.

Human activity on our planet has caused global changes of atmospheric chemistry and climate. These changes are likely to amplify in the near future due to increasing concentrations of greenhouse gases such as carbon dioxide (CO_2_) in the atmosphere, and will result in increased temperature and altered precipitation patterns including more intense summer droughts over large parts of the Earth’s surface^1^,^2^. Such profound shifts of atmosphere chemistry and climate will fundamentally affect key drivers of ecosystem functioning and lead to changes in terrestrial ecosystems across the globe^3^,^4^. Each of these changes may affect ecosystem functioning, e.g. through CO_2_-stimulated photosynthesis and plant growth^5^, warming-induced increase in nutrient mineralization^6^,^7^ or drought-induced mortality and growth limitation of plants and animals^8^,^9^. The number of such single-factor studies has grown considerably, but studies considering simultaneous action of multiple climate change drivers are still relatively sparse. However, understanding their co-action on terrestrial ecosystems is of paramount importance in order to increase accuracy of global change predictions^10^,^11^. Aboveground responses to climate change have been more comprehensively studied in terrestrial ecosystems, whereas belowground organisms and processes have received less attention. There is an urgent need for obtaining insights into the mechanisms and patterns of belowground biota response to climate change, and especially quantifying combined action of different climate change agents.

Elevated CO_2_ levels are unlikely to have direct negative effects on belowground organisms, since soil biota are adapted to much higher CO_2_ concentrations in the soil pore space than found in the atmosphere. Most studies have shown that increased atmospheric CO_2_ concentrations affect soil biota positively^12^, probably due to increased primary production^13^, which would lead to increased litter input, root growth and root exudation, the major food sources for decomposer food webs^14^,^15^. Moreover, elevated CO_2_ improves the water use efficiency by plants and hence potentially increase the soil water content. Such conditions are also beneficial for most soil organisms^16^,^17^.

Increased temperature is likely to extend the growing season of plants in temperate regions with additional positive effects on root productivity, microbial biomass and activity^11^,^18^,^19^,^20^. Although such changes could potentially lead to increased food supply relevant for various soil fauna groups, most studies suggest that moderate temperature increase ascribed to climate change in the near future will have little impact on soil fauna^12^,^21^,^22^,^23^.

Increased occurrence and intensity of summer droughts may induce dramatic effects on various soil biota groups. This is especially true for soil invertebrates dependent on free water in the soil pores. Thus, enchytraeids are sensitive to decreased water content in soil, whereas microarthropods are probably more resistant to drought^16^,^24^,^25^,^26^,^27^,^28^. Apart from the direct effects of drying, negative effects of drought on plant growth and root exudation may also lead to reduced food availability for many decomposer organisms^12^.

Changes in the environmental conditions as described above may improve or worsen the chances for survival and growth of each species’ population in the given ecosystem and consequently change diversity status of the whole belowground community. There is growing evidence of the link between biodiversity of soil fauna and the level of ecosystem services delivery. This is particularly true for carbon sequestration and cycling^29^,^30^,^31^. It is therefore important from the perspective of belowground community functionality to evaluate the impact of climatic changes on soil fauna biodiversity^12^,^32^.

Recently, significant advances in our understanding of the causality of shifts in belowground ecological processes associated with environmental changes have been achieved by the use of Structural Equation Modeling (SEM) that allows parameterizing of complex models with many parameters and latent variables^33^,^34^,^35^. For example, the effects of drought, increased CO_2_ and nitrogen addition on soil food webs have been analyzed using SEM^10^,^36^.

In this study we quantified the simultaneous co-action of multiple environmental drivers usually associated with climate change on the structure and functionality of belowground fauna. In a field trial, we exposed a semi-natural Danish heathland to elevated atmospheric CO_2_ concentration (CO_2_), extended summer drought (drought) and increased temperature (warming) and their combinations in a full factorial experimental setup. The full combination of these treatments aimed to mimic climate predictions for Denmark year 2075 as closely as possible^37^. In addition to a comparison of un-treated control and the full combination of treatments, we used SEM analysis to investigate the effects of the climate treatments on the biodiversity (using the Shannon index) of various soil invertebrate groups, i.e. the interrelations between taxonomic or functional diversity of major soil invertebrate taxa (nematodes, enchytraeids, collembolans and oribatid mites), quality of their putative food sources and the main abiotic drivers (soil temperature and water content). We analyzed how the three manipulated factors have influenced biodiversity after two years of treatment, and after eight years of treatment, respectively. We hypothesized that (i) elevated CO_2_ would increase litter C:N ratio, fungi:bacteria ratio and microbial C:N ratio, which in turn would increase biodiversity of soil fauna through trophic interactions, (ii) reduction of soil water content would reduce biodiversity, and (iii) that moderately increased temperatures would have no direct effects on biodiversity of soil biota, but decrease the soil water content and therefore indirectly decrease biodiversity of soil invertebrates. Finally, (iv) we expected that climate change effects on soil biota were persistent and increasing with time, in particular for organisms with long generation times.

## Results

### Soil fauna communities

The nematode community was dominated by bacterial feeders (in particular belonging to the genera *Acrobeloides, Cervidellus, Monhystrella, Plectus* and *Teratocephalus*; data not shown) and plant parasitic species (especially the genera *Paratylenchus* and *Helicotylenchus*; data not shown). Fungal feeders (mostly the genera *Aphelenchoides* and *Tylencholaimus*; data not shown) and carni-omnivorous species (dominated by the genus *Aporcelaimellus*; data not shown) were less abundant (Table S1).

The enchytraeid community at the field site was dominated by *Chamaedrilus chlorophilus* which represented about 75% of the total population (Table S2). Three other species, *Achaeta affinis, Oconnorella cambrensis* and *Enchytronia parva* were also relatively abundant, and eight other species occurred sporadically.

We identified about 20 species of Collembola (Table S3a,b). The community was dominated by euedaphic and hemiedaphic species such as *Mesaphorura sp., Megalothorax minimus, Parisotoma notabilis*, and *Protaphorura sp.* Larger epedaphic species (e.g. *Tomocerus sp.* and *Orchesella sp.*) did occur, but were much less abundant. Species richness of Collembola was similar in 2007 and 2013 (Table S3a,b).

About 35 species of oribatid species were recorded in 2007 (Table S4a). The most abundant species were found among the genera *Galumna, Microppia, Scheloribates* and *Suctobelbella*. Species richness of oribatid mites was higher in 2013 than in 2007, mainly due to the appearance of numerous oribatids from the Brachychthoniidae family (Table S4b).

### Structural Equation Modeling

**Figure 1:**
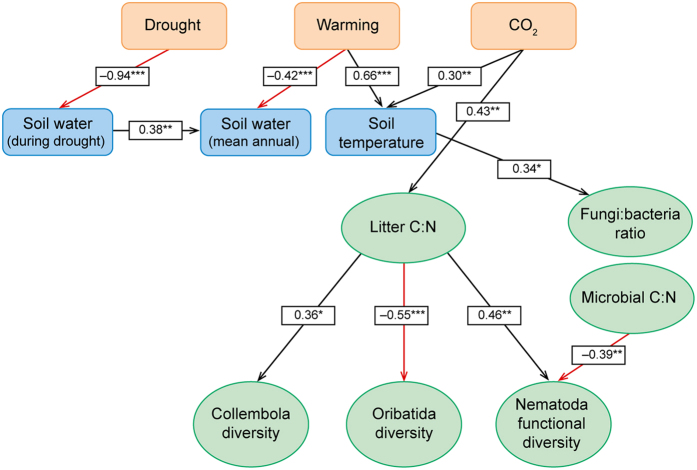
Structural equation model of effects of elevated CO_2_, drought and warming (exogenous factors; pink boxes) on physical soil parameters (blue boxes) and on litter C:N ratio, fungi:bacteria ratio, soil microbial C:N ratio and diversity (Shannon index) of Collembola, Oribatid and Nematoda communities (green ovals) in a Danish heathland exposed to two years of climate change manipulation. Litter, microbial parameters and fauna was sampled in autumn 2007. Numbers on arrows are standardized regression coefficients. Regression coefficients marked with ** or *** are highly significant (*P* < 0.01 and *P* < 0.001, respectively) and coefficients marked with * are significant (0.01 < *P* < 0.05) with *N* = 48; black arrows indicate positive relationships, red arrows indicate negative relationships. Non-significant correlations are not shown (full model is shown in Table S5a).

**Figure 2:**
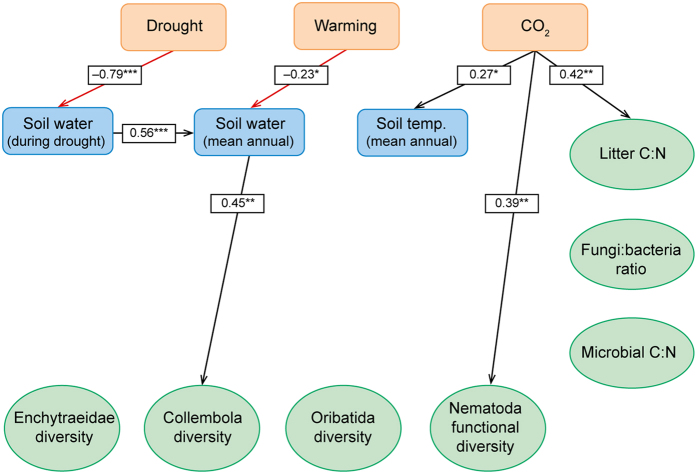
Structural equation model of effects of elevated CO_2_, drought and warming (exogenous factors; pink boxes) on physical soil parameters (blue boxes) and on litter C:N ratio, fungi:bacteria ratio, soil microbial C:N ratio and diversity (Shannon index) of Collembola, Oribatida, Enchytraeidae and Nematoda communities (green ovals) in a Danish heathland exposed to eight years of climate change manipulation. Litter, microbial parameters and fauna was sampled in April 2013. Numbers on arrows are standardized regression coefficients. Regression coefficients marked with ** or *** are highly significant (*P* < 0.01 and *P* < 0.001, respectively) and coefficients marked with * are significant (0.01 < *P* < 0.05) with *N* = 48; black arrows indicate positive relationships, red arrows indicate negative relationships. Non-significant correlations are not shown (full model is shown in Table S5b).

The significant relationships of the full model for the two years are shown graphically in Figs 1 and 2 and the summary statistics of the fitted structural equation models for the observations made two (October 2007) and eight years (April 2013) after the start of the experiment are shown in Table S5a and b, respectively. The fit of the SEM converged normally and the fitting properties were assessed using the standardized root mean square residual statistic (SRMR) which range from zero to one, where zero indicates a perfect fit and 0.08 is judged to be acceptable^38^. The SRMRs were estimated as 0.06 and 0.080 in 2007 and 2013 (*N* = 48 in both years), respectively, and consequently the fit was judged to be acceptable.

Since the model was based on prior ecological knowledge of the causal relationships we did not perform any model reduction of non-significant relationships. This choice was made in order not to perturb the *a priori* specified partitioning of variance in the structural equation model by committing type II errors due to insufficient sample sizes. In other words, if an arrow is falsely removed due to a relatively low sample size, then the variation explained by the removed arrow is unjustifiably “forced” into another causal pathway.

### Soil water content and temperature

The three exogenous variables, i.e. the CO_2_, warming and drought treatments, generally had significant effects on the measured soil temperature and soil water content of the plots (Figs 1 and 2; Table S5a,b). As expected, the drought and warming treatments both had significant negative effects on soil water content in both years. The warming treatment had a positive effect on soil temperature, however, the effect was statistically significant only in 2007. CO_2_ had a significant positive influence on soil temperature in both years although the effect was not large (about 0.1 °C; Table S6). It is difficult to give a mechanistic explanation of this effect, but it is hardly a realistic consequence of climate change and perhaps rather an artefact of the experimental setup. For 2007 data, the model explained 89% of the variation in soil water content during drought, 32% of mean annual soil water content, and 52% of the variation in soil temperature (see R^2^-values in Table S5a). For 2013 data, the model explained 63% of the variation in soil water content during drought, 37% of mean annual soil water content, but only 13% of the variation in soil temperature (Table S5b).

### Litter and microbial parameters

The litter C:N ratio was significantly increased by elevated CO_2_ (models explained about 20% of variation in both years), but was not affected by any other variable (Table S5a,b). The fungi:bacteria ratio was significantly correlated with soil temperature in 2007 with 17% of the variation explained by the model, but this correlation had disappeared in 2013. The microbial C:N ratio was not influenced by CO_2_, soil water content or soil temperature. The litter C:N was higher in October 2007 than in April 2013, probably due to the different seasons when samplings took place (Table S7). The fungi:bacteria ratio was lower in 2007 than in 2013 and was not correlated with CO_2_ or other variables (Figs 1 and 2; Table S7).

### Biodiversity

**Figure 3:**
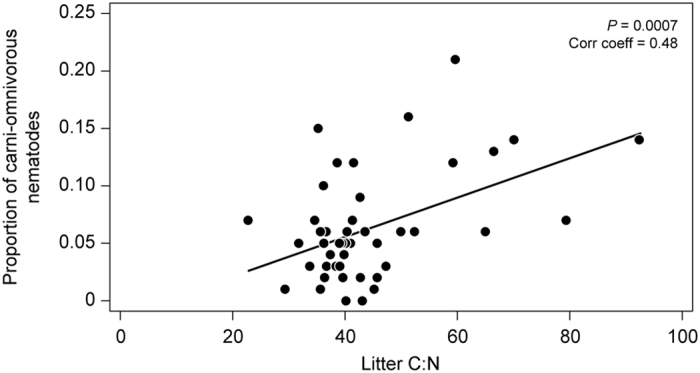
Proportion of carni-omnivorous nematodes plotted against litter C:N ratio (October 2007). Solid line indicates a significant positive correlation (*P* = 0.0007; *N* = 48).

The nematode functional diversity (i.e. the calculated Shannon index using feeding groups as categories) was significantly and positively correlated with litter C:N, and negatively correlated with microbial C:N in 2007. A substantial part of the variation in nematode functional diversity was explained by the model (39%). The positive correlation with litter C:N was mainly driven by increased abundance (data not shown) and proportion of carni-omnivorous nematodes at high litter C:N (Fig. 3) without an increase in the total nematode population (data not shown). The negative correlation between nematode functional diversity and microbial C:N in 2007 was not explained by the abundance of any particular feeding group (Table S1). In 2013, there was a significant correlation of nematode functional diversity with CO_2_, but correlations with litter C:N or microbial C:N had disappeared. Moreover, the model explained only 24% of the variation in nematode functional diversity, i.e. much less than in 2007, and the effect of CO_2_ was relatively small (Table S1).

In 2007, biodiversity of collembolans correlated with litter C:N, but this correlation was not present in 2013 (Figs 1 and 2). In 2013, we found a significant (positive) correlation between the biodiversity of collembolans and mean annual soil water content (Fig. 2). This effect seemed to be associated with a decrease of epedaphic species at the lower soil water contents, but the model explained only a minor part of the variation of collembolan diversity (16%; Table S5b).

**Figure 4:**
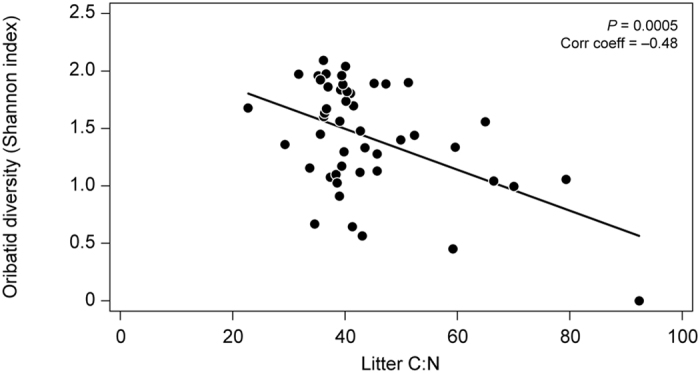
Shannon diversity index of oribatid mite communities in 2007 plotted against litter C:N ratio. Solid line indicates a significant negative correlation (*P* = 0.0008; *N* = 48).

In 2007, the biodiversity of oribatids was negatively correlated with litter C:N (Fig. 1), and the model explained 31% of the variation (Table S5a). The effect of litter C:N was quite strong with decreases of about 40% of biodiversity on average across the range of litter C:N values (Fig. 4). It was, however, not possible to identify particular species that were responsible for this relationship (Table S4a). In 2013 (Table S4b), we found no significant correlations between the biodiversity of oribatids and any of the explanatory variables (Fig. 2; Table S5b). The biodiversity of the enchytraeid community was not assessed in 2007, and in 2013 it did not correlate with any physical or biological parameters of the model (Fig. 2).

**Figure 5:**
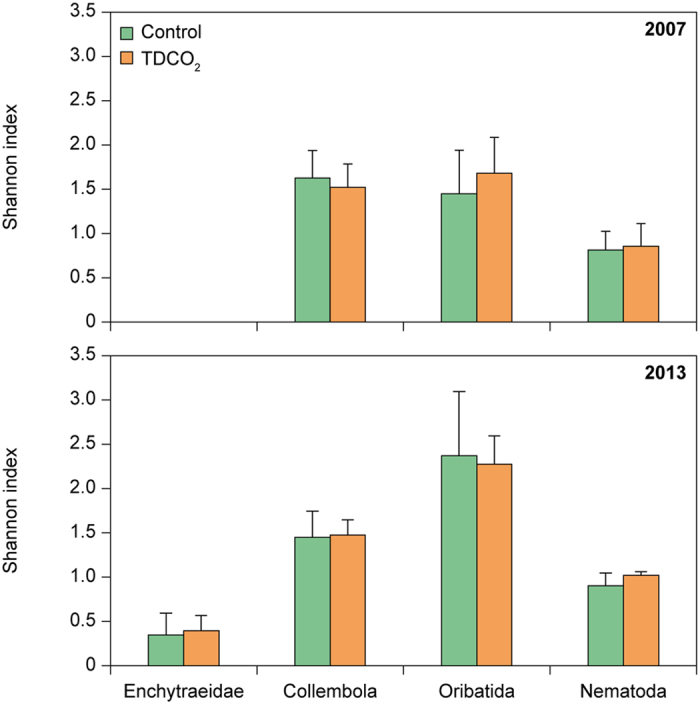
Shannon diversity indices (mean ± s.d.; *N* = 6) in October 2007 (upper panel) and April 2013 (lower panel) of Enchytraeidae (only in 2013), Collembola, Oribatida and functional diversity of Nematoda (based on feeding groups) for ambient plots (control; green columns) and plots receiving a combination of elevated CO_2_, increased drought and warming (TDCO_2_; orange columns). No significant differences between treatments were found.

In addition to the SEM analysis we compared biodiversity of ambient plots and plots that had received the full combination of treatments for eight years (i.e. TDCO_2_). Using a one-way ANOVA we noted that TDCO_2_ did not differ significantly from ambient plots for any of the diversity indices after two years or after eight years of treatments (Fig. 5).

## Discussion

It is quite unique to have detailed information on diversity within several groups of soil organisms rich in species as well as in individuals. To our knowledge it is the first time that data on diversity of nematodes, enchytraeids, collembolans, and oribatid mites are simultaneously analyzed in a multifactorial global change experiment. Moreover, by performing this detailed assessment of organism diversity twice (two and eight years after field manipulations started) we can analyze how stable the global change effects on soil biota are over a longer time scale. The fauna groups studied have small body sizes and relatively low mobility and we judge field plots to be sufficiently large to avoid noteworthy emi- or immigration and edge effects from neighbouring plots receiving a different treatment.

We stated earlier that each of the exogenous factors to some extent had unintentional influence on physical conditions of soil, hence precluding a traditional general linear model (nested) ANOVA approach where the treatments are assumed to be orthogonal. Indeed, we observed that experimental warming caused a significant (albeit not very pronounced) reduction of soil water content. The effect of warming causing decreased soil moisture was, however, apparently not related to increased temperature (and thus evaporation) *per se*, but probably more related to reduced dew formation during night-time due to the reflective curtains used for night-time passive warming. Lastly, FACE had a slight, but significant effect increasing the soil temperature. These observations further justify the SEM approach.

Other studies in which temperature have been manipulated more efficiently than in our study have demonstrated that warming can have influence – most often negative - on abundance and diversity of various soil fauna groups^12^,^22^,^39^. It has been proposed that warming favors short-lived and fast-reproducing species more than longer-lived species resulting in altered community composition and diversity^40^. The warming treatment in our study had limited effect on mean soil temperature (less than 1 °C on average) which may be a major reason why we did not observe direct effects of temperature on biodiversity. However, the SEM modeling indicated that experimental warming had an indirect negative effect on the biodiversity of collembolans through reduced soil water content.

The increase in the diversity and abundance of oribatid mites from October 2007 to April 2013 may be the result of seasonal fluctuations rather than a general increase of diversity over this time period. The high proportion of Brachychthoniidae and Oppiidae species in April 2013 may be due to massive hatching of their eggs during the well-known spring-peak of oribatid abundance^41^. However, the recorded differences in the overall abundance between two sampling occasions are within the known range of abundance and diversity variance across different seasons and years^42^,^43^.

Several other field studies similar to ours have shown that reduction of precipitation significantly decreased belowground biodiversity and abundance of soil fauna^12^, but some of these studies tended to have used more dramatic reductions of precipitation^44^,^45^ than we have. We note that drought intensity did not have direct influence on biodiversity when assessed months after the end of dry conditions, whereas we did observe an effect of mean annual soil water content on the diversity of collembolans. Our previous studies have shown that drought can cause substantial and acute negative effects on abundance of nematodes, collembolans and enchytraeids in our study area and similar ecosystems^25^,^36^,^46^,^47^. Biomass of enchytraeids, as determined shortly after experimental drought episodes, was reduced when water content (0–20 cm depth) was approaching 5 volume %, but the population recovered within months after the soil water content was restored^25^,^36^,^48^. The enchytraeid community at the study site was heavily dominated by *C. chlorophilus*, but about ten other species were also recorded. Despite severe reduction in numbers and biomass during droughts, we did not detect significant correlations between soil moisture and biodiversity of the enchytraeid community (neither soil water content during drought, nor annual mean water content). This may indicate that the enchytraeid community present at the study site is well adapted to occasional drought episodes, but it cannot be ruled out that some species preferring moist or wet conditions in the long run will become reduced in abundance or completely disappear in a drier environment as shown in another long-term climate change experiment^9^.

It has been reported that reduced stomatal conductance of plants under CO_2_ enrichment is most significant under drought regimes^49^ and that this may lead to a better water use efficiency and lessened depletion of soil water. In the present experiment we found no evidence that increased aerial CO_2_ concentrations led to increased soil moisture in the experimental plots. Thus, the decreased biodiversity of collembolans in dryer soils observed in 2013 was not related to CO_2_ enrichment, but rather related to legacy of the experimental drought periods and a small, but consistent, drying effect of the warming treatment. The positive correlation between soil water content and biodiversity of collembolans in 2013 was mainly driven by an increase in species richness (linear regression; *P* = 0.02; data not shown). The biodiversity of oribatids was not influenced by soil moisture in our experiment, perhaps due to their higher desiccation resistance as compared to other fauna groups^26^, and that our drought treatment was moderate. Studies in which drought was longer-lasting show that also oribatid communities can be severely impacted by drought and that recovery of biodiversity after severe drought incidents can take several years due to the relatively slow reproduction of these animals compared to other microarthropods such as collembolans^44^,^45^,^50^.

Despite the lack of an overall effect of the future climate scenario (i.e. TDCO_2_) on biodiversity, we were able to detect separate effects of increased CO_2_ (after two years of treatments) on biodiversity of oribatid mites and collembolans, and on functional diversity of nematodes. Increased atmospheric CO_2_ increased the C:N ratio of litter, and decreased the biodiversity of oribatids downstream, whereas diversity of collembolans increased. Elevated CO_2_ also stimulated abundance of long-lived and carni-omnivorous nematodes, and thus functional diversity of nematodes, likely due to an increased root biomass and root exudation which in turn increased the biomass of microbes and prey that this group of nematodes is feeding on. In 2013, elevated CO_2_ still affected nematode functional diversity, but, interestingly, at this point the CO_2_ effect was independent of litter C:N ratio. Nematodes are to a larger degree associated with the rhizosphere than oribatids, collembolans or enchytraeids and therefore more responsive to quantitative and qualitative root exudation CO_2_ responses. The litter independent CO_2_ effect on nematode functional diversity may therefore reflect increased root exudation at elevated CO_2_ as reported elsewhere^51^,^52^.

We detected no effects of CO_2_ on enchytraeid biodiversity although litter chemistry and other food-related parameters were clearly affected by CO_2_ with imprints on the physiology of enchytraeids at our field site^36^,^46^. Thus, a previous study of N-content of soil fauna at the same field experiment showed that elevated CO_2_ reduced enchytraeid nitrogen concentration, whereas collembolans were not affected, and oribatid mites had higher nitrogen concentration than at ambient CO_2_^36^. This may reflect differences in the feeding biology between oribatid mites, enchytraeids and other soil fauna taxa, and indicates that aerial CO_2_ enrichment can have direct or indirect influence on animal nutritional physiology which may potentially manifest itself at higher levels of biological organization such as species abundance and diversity. This suggests that at elevated CO_2_, soil animals tend to be more nitrogen-limited and switch to alternative food resources which are N-enriched, e.g. shifting from direct detritus to predominantly fungal or bacterial feeding.

Despite the fact that we could identify significant separate effects of soil moisture and CO_2_ on soil animals, we saw no differences in biodiversity of any focal soil fauna taxon between ambient plots and plots receiving the full combination of treatments representing a likely climatic scenario for Denmark in year 2075. This reveals a pronounced resistance potential of belowground communities to the upcoming climatic shifts which might be explained by the high thermal and hygric buffering capacity of soil^53^ and redundancy of soil food webs^32^,^54^. Our study of effects of a persistent global change on soil biota over a period from few to many generations of the longest living members is quite unique. Reported recovery of soil fauna communities after disturbances show various levels of resilience ranging from decades after mining activity^55^ or years after extended drought^50^, to months following burning^56^,^57^,^58^ and moderate drought^59^,^60^.

The SEM performed on the same type of data obtained two and eight years after initiating global change manipulations showed that interactions from experimental manipulations via soil physical environment and litter chemistry to soil biota were markedly reduced over this period. The six significant interactions observed after two years were six years later reduced to only three significant interactions. The CO_2_ effect on litter C:N persisted for eight years of manipulation, however, litter C:N effect on diversity of oribatids, collembolans and nematodes after two years had disappeared after eight years. Eight years since the experiment started, the diversity of collembolans and nematodes depended directly on soil moisture and CO_2_ treatment, respectively. This was not what we expected and suggests that soil fauna has a marked resilience initially being strongly affected by abrupt environmental changes, and then later adjusting to the pre-manipulation state.

Although it could be argued that the present ecosystem did not have a high biodiversity to begin with, it is remarkable that species composition within four taxa belonging to the soil food web is only in the short term affected by a stable global change-induced shift in litter chemistry. When global change in this study did not have permanent effect on composition of the soil fauna, the important question that appears is: which factors are drivers of changes in soil fauna diversity? Although soil faunal diversity was only temporarily affected by the projected global change parameters, long-term global change did affect the functionality of the soil at the field site. For instance, labile carbon-induced soil organic matter decomposition (priming) was enhanced after long-term elevated CO_2_ treatment^61^. Hence, the resilience of soil biotic diversity should not lead us to conclude that in the future climate change scenario soil functionality will not be affected. Further, in a larger context, our study represents merely one particular future climate scenario in a local environment. We must therefore be cautious in attempting to extrapolate our findings to larger regions and climate change scenarios. However, the very clear result of our study suggests that belowground biodiversity can be quite resilient in responding to a realistic climatic scenario. Moreover, we detected separate effects of increased CO_2_ and soil moisture on the biodiversity of soil fauna, but these effects were not detected when the treatments were combined.

## Methods

### Site description and experimental design

**Figure 6:**
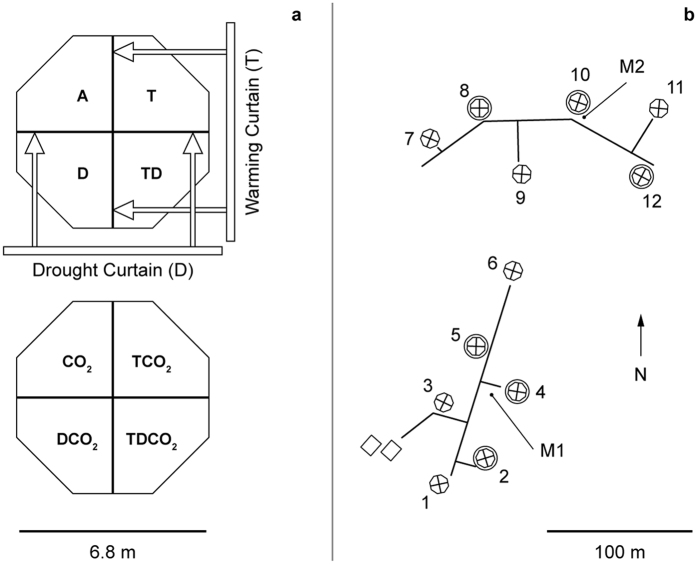
(**a**) The layout of a single block of the experiment. The direction of extension of the curtains that provided the night-time warming (T) and drought (D) treatments is shown on the upper octagon. One octagon is at ambient [CO_2_] (380 ppm) while the other received elevated [CO_2_] (510 ppm) from the Free Air Carbon Enrichment (FACE) system. (**b**) The arrangement of the field experiment at Brandbjerg, Denmark. The octagons are labelled 1 through 12, with consecutive pairs belonging to the same experimental block. Octagons 2, 4, 5, 8, 10 and 12 received elevated [CO_2_] and are circled. The boardwalks are shown by solid lines and the locations of two meteorological stations are shown as M1 and M2. The two rectangles represent buildings that housed computers, control systems and field laboratories.

The experimental site was a heath/grassland ecosystem dominated by grass (*Deschampsia flexuosa*) and an evergreen dwarf shrub (*Calluna vulgaris*). The soil was a coarse textured sandy arenosol from the Weichsel glaciation with only weak signs of podsolization. The annual mean temperature (1961–1990) of this area (Brandbjerg, Denmark; 55°30′ N, 11°80′ E), was 8.0 °C and the annual mean precipitation amounted to 613 mm^37^. The experiment was designed to mimic a realistic climate scenario for Denmark in year 2075, with elevated atmospheric CO_2_ (CO_2_), warming (T) and extended summer drought (D). Atmospheric CO_2_ concentration was increased during daytime hours in a Free Air CO_2_ Enrichment (FACE) setup (ambient (380 ppm) + 130 ppm = 510 ppm). Warming was done using automatic, passive night-time warming curtains, which overall for the period 2006–2013 increased mean monthly night-time temperatures in 20 cm height by 0.4 and 1.1 Kelvin during winter and summer months, respectively. In 5 cm soil depth, the corresponding increases were 0.17 and 0.35 Kelvin, respectively. Although these temperature increases were moderate it significantly increased the number of degree days in warmed plots (by 30 to 70 degree days in soil, and mostly during the growth season) during all experimental years (Fig. S1). The drought treatment was active during a 4–6 week campaign each year usually during early summer using automatic rainout curtains removing 5–11% of annual precipitation. The experimental setup was full factorial in a split-plot design, i.e. including all possible combinations of the three treatments. All combinations of treatments were replicated 6 times (i.e. 2^3^ treatments × 6 replicates; *N* = 48). T and D treatments were nested within the CO_2_ treatment, i.e. six octagons (6.8 m diameter) were treated with elevated atmospheric CO_2_ while six octagons had ambient CO_2_ conditions (A). Each octagon was divided into four ‘slices’ (9.1 m^2^ per plot) to provide all eight treatment combinations in each pair of octagons (Fig. 6). See also Mikkelsen *et al*.^37^ for further details. We did not use the treatments as such, but the derived moisture and temperature effects in the SEM analysis (see later description of statistical analysis). The plots contained a mixture of patches with *Deshcampsia* and *Calluna*, but our investigations here consider only areas dominated by *Deschampsia*. In order to reduce edge effects as much as possible soil samples were taken at random, but at least 50 cm from the edge of the plot. The distances between the octagons were at least 2.5 times the octagon width to avoid CO_2_ contamination from the elevated to the ambient CO_2_ octagons. Boardwalks connected the octagons to avoid disturbance by trampling between the plots and a flexible boardwalk system in each octagon provided easy access and prevented trampling within the plots. Within each experimental octagon or plot, parameters were monitored to check the treatments and their effects on the physical and climatic conditions. Temperature data used in this study were measured in the soil (–5 cm) in each treatment plot every minute and averaged over hourly intervals. Soil moisture was measured by TDR at 0–20 cm soil depth providing half-hour averages.

### Explanatory variables

We employed SEM allowing parameterizing relations between environmental parameters and biodiversity of various soil animal groups. Thus, two of the three applied treatments (warming and drought) were not entered as categorical variables; instead we analyzed how treatments influenced soil temperature and moisture directly and used these parameters as explanatory variables. As discussed above, the applied treatments (exogenic factors) sometimes had indirect effects on physical conditions in the soil (e.g. CO_2_ increased soil temperature; warming decreased soil water content) hence confounding a traditional general linear model ANOVA approach. We therefore calculated plot-specific mean soil temperature (5 cm depth) during the 12 month period preceding sampling of biotic parameters in October, 2007, and in April, 2013, respectively. Likewise, plot-specific mean soil water contents (mean SWC) were calculated for the same periods. Finally, mean soil water content during the last week of the latest applied drought period before sampling of biotic parameters (June 15–22, 2007, and June 4–11, 2012, respectively; mean drought SWC) were used as a measure of the intensity of the latest drought application. A summary of these explanatory variables are shown in Supplementary Information Table S6.

### Measurements of biotic components

Measurements of several biotic components were made in October 2007, about two years after climate treatments began in October 2005. Measurements were repeated in April 2013, eight years after treatments began. Some of these results have been published elsewhere (see Supplementary Information), but a brief description of the sampling methodology follows here.

### Nematodes

Nematodes were extracted from between 10 and 40 g (fresh weight) of soil by a modified Baermann tray extraction method^62^. Samples were extracted for 48–72 h, and nematodes were, based on the morphology of mouthparts and the oesophagus^63^, then classified to different trophic groups (predacious, omnivorous (these two groups were grouped as carni-omnivorous), herbivorous, fungivorous and bacterivorous) and counted at x40 magnification using a dissecting microscope. Based on the relative abundances of these trophic groups we calculated the Shannon index as a measure of “functional diversity” of the nematode community.

### Enchytraeids

Enchytraeids were sampled using a cylindrical soil corer with an inner diameter of 5.5 cm to a depth of 9 cm. Each soil core was divided into layers 3 cm thick with a knife. The samples were kept in plastic beakers at 5 °C until extraction which was initiated within two weeks after collection. The extraction was a modified version of O’Connor’s wet funnel extraction^64^ performed at room temperature over 6 hours. Extracted enchytraeids were identified *in vivo* based on the key and the handling procedure described in Schmelz and Collado^65^. Based on the relative abundances of species we calculated the Shannon index for enchytraeids.

### Microarthropods

Microarthropods were sampled using a cylindrical soil corer with the inner diameter of 6 cm to a depth of 10 cm. Each soil core was divided in the field into two layers, each 5 cm thick with a knife. The samples were kept in closed plastic cylinders at 5 °C until extraction which was initiated within two weeks after collection. They were extracted in a high gradient extraction apparatus (MacFadyen type), where the temperature in the upper compartment increased stepwise from 25 °C to 50 °C within seven days while the temperature at the lower compartment remained constant at 3 °C^66^. The microarthropods were collected in benzoic acid and subsequently conserved and stored in glycerol until identification. Collembolans were identified to species level according to Fjellberg^67^,^68^. Oribatid mites were identified to species or genus level as described in detail by Zaitsev *et al*.^69^. Based on the relative abundances of species we calculated the Shannon index for Collembola and Oribatida, respectively.

### Fungi:bacteria ratio, litter and microbial C:N ratio

Responses in biodiversity of fauna was related to the quality of the litter input and microbial biomass by assessing the fungi:bacteria ratio, and the C:N ratio of the soil microbial biomass and litter in each plot. Fungi:bacteria ratio was based on copy numbers of the internal transcribed spacer (ITS) region of fungal ribosome-encoding genes, and the bacterial 16S rRNA gene, respectively, as quantified by qPCR. Details of the method are published elsewhere^70^. Soil microbial C:N ratio was determined as follows. Fresh soil from each plot was extracted directly, and a parallel soil sample after vacuum-incubation, with chloroform for 24 hours^71^,^72^. A digestion with H_2_SeO_3_, H_2_SO_4_ and H_2_O_2_ was followed by the NH_4_^+^ analysis (indophenol-blue reaction). Total microbial N (MicN) was calculated as N in the fumigated sample minus N in the non-fumigated sample, using 0.4 as the extractability factor^73^. Analysis for carbon (dissolved organic, DOC) was done using a Shimadzu TOC 5000A analyzer (Shimadzu, Kyoto, Japan). Total microbial C (MicC) was calculated as DOC in the fumigated sample minus DOC in the non-fumigated sample, using 0.45 as the extractability factor^74^. Microbial C:N ratio was MicC divided by MicN. Plant litter was sampled in September-October 2007 and in June 2013 in mixed vegetation. The air dried litter was analyzed for total C and N by Dumas combustion (1020 °C) on an elemental analyzer (EA Flash 2000, Thermo Fisher Scientific, Milan Italy).

### Structural equation modeling

In structural equation models (SEM) a hypothesis of the causal relationships between the measured variables in a system is fitted to observed data^34^. The main use of an SEM is to quantify which of possible direct or indirect causal pathways that is most important in regulating a variable, and whether the influences of different factors on a variable are positive or negative. Generally, a SEM does not prove the hypothesized causal relationships, but it is possible to test if specific causal relationships are supported by data. The measured abiotic and biotic variables were arranged in a causal network according to prior knowledge of soil ecological cause-effect relationships (Figs 1 and 2; Table S5a,b). The prior ecological knowledge was specified by applying generally established ecological theory that for many of the specific causal relationships had been studied at the experimental site^36^,^46^. Since the measurements made in 2007 and in 2013, and the number of replicates (*N* = 48), were largely the same, we had the possibility to compare and infer from data the appearance of resilience over time. Some of the variables were log-transformed in order to achieve an approximate normal distribution and some of the variables were rescaled so that all variables had an approximately equal scale. From graphical inspection of the distribution of the variable, it was judged whether or not to log-transform the variable. After rescaling, the variables were fitted to the structural equation model using the software package lavaan^75^ with all the nodes and edges of the prior model. In order to increase precision and take into account the effect of covariance between soil temperature and soil water content, the measured environmental states of the plots were used to predict soil fauna diversity rather than the exogenous treatment levels (0 or 1) of temperature and drought.

## Additional Information

**How to cite this article:** Holmstrup, M. *et al*. Long-term and realistic global change manipulations had low impact on diversity of soil biota in temperate heathland. *Sci. Rep.* **7**, 41388; doi: 10.1038/srep41388 (2017).

**Publisher's note:** Springer Nature remains neutral with regard to jurisdictional claims in published maps and institutional affiliations.

## Supplementary Material

Supplementary Information
