# Supplementary material for: Long-term and realistic global change manipulations had low impact on diversity of soil biota in temperate heathland
Source: Sci Rep. 2017 Jan 25;7:41388. doi: 10.1038/srep41388 (PMC5264592; doi:10.1038/srep41388)
Supplement: Supplementary Information [file srep41388-s1.pdf]

Supplementary Information for

"Long-term and realistic global change manipulations had low impact on diversity of soil biota in temperate heathland"

authored by:

Martin Holmstrup[1,2]*, Christian Damgaard[1], Inger K. Schmidt[3], Marie F. Arndal[3], Claus Beier[3], Teis N. Mikkelsen[4], Per Ambus[5], Klaus S. Larsen[3], Kim Pilegaard[4], Anders Michelsen[6], Louise C. Andresen[7], Merian Haugwitz[6,8], Lasse Bergmark[8], Anders Priemé[8], Andrey S. Zaitsev[9,10], Slavka Georgieva[6,11], Marie Dam[6], Mette Vestergård[6], Søren Christensen[6]

[1] Department of Bioscience, Aarhus University, Vejlsøvej 25, 8600 Silkeborg, Denmark
[2] Aarhus Institute of Advanced Studies, Aarhus University, Høegh-Guldbergs Gade 6B 8000 Aarhus C, Denmark
[3] Section for Forest, Nature and Biomass, Department of Geosciences and Natural Resource Management, University of Copenhagen, Rolighedsvej 23, 1958 Frederiksberg, Denmark
[4] Department of Environmental Engineering, Atmospheric Environment, Technical University of Denmark, 2800 Kgs. Lyngby, Denmark
[5] Section for Geography, Department of Geosciences and Natural Resource Management, University of Copenhagen, Øster Voldgade 10, 1350 Copenhagen, DK
[6] Terrestrial Ecology Section, Department of Biology, University of Copenhagen, Universitetsparken 15, 2100 Copenhagen Ø, Denmark
[7] Department of Earth Sciences, University of Gothenburg, P.O. Box 460, 405 30 Gothenburg, Sweden
[8] Section of Microbiology, Department of Biology, University of Copenhagen, Universitetsparken 15, 2100 Copenhagen Ø, Denmark.
[9] Institute of Animal Ecology, Justus-Liebig-University, Heinrich-Buff Ring 26-32 (IFZ), 35392 Giessen, Germany
[10] A.N. Severtsov Institute of Ecology and Evolution, Russian Academy of Sciences, Leninsky prospekt, 33, Moscow 119071, Russian Federation
[11] Department of Zoology and Anthropology, Faculty of Biology, Sofia University, Dragan Tzankov 8, 1164 Sofia, Bulgaria

* Corresponding author: Martin Holmstrup; email: martin.holmstrup@bios.au.dk
phone: +45 3018 3152

Table S1. Mean abundance of nematodes (m^{-2}) belonging to different feeding groups and the Shannon index of functional diversity (nematodes classified by feeding groups) in ambient (A; control), warmed (T), drought (D) and CO_2 treated plots (CO2) at the CLIMAITE field site.

	A		T		D		TD		CO2		TCO2		DCO2		TDCO2	
	mean	SD	mean	SD	mean	SD	mean	SD	mean	SD	mean	SD	mean	SD	mean	SD
2007																
Bacterial feeders	103505	52331	135420	111593	85233	42768	113100	60660	148115	91946	81137	32435	125826	103816	100693	36779
Fungal feeders	6587	3920	3317	3638	3794	4533	8448	8010	2789	2475	6654	4866	5577	8395	6514	4470
Plant parasitic	44211	23959	42275	39339	36929	25310	38209	17486	69110	63100	49804	28636	71865	93864	40564	30414
Omnivorous	4625	4238	6428	7282	7119	3958	6012	3508	12705	4445	12372	4476	11259	3798	9283	6289
Predacious	0	0	1360	3331	463	827	1097	1800	1013	1295	3221	5011	177	433	1473	3608
Shannon index of diversity*	0.82	0.21	0.71	0.29	0.89	0.11	0.87	0.12	0.85	0.18	1.03	0.18	0.93	0.14	0.86	0.26
2013																
Bacterial feeders	85931	53409	52093	26456	85709	58830	74902	81811	69849	50369	43072	46107	75140	61313	105208	61487
Fungal feeders	24993	24121	23710	25868	19556	9862	32357	51361	103955	111658	21329	19090	81898	100422	31450	12523
Plant parasitic	95939	54474	91415	72014	58456	36454	61095	41061	106725	107172	56092	32302	73256	67750	111261	75492
Omnivorous	1631	1710	4832	5145	1823	1697	4311	4216	6009	3155	2037	3814	5022	7104	4715	4853
Predacious	0	0	128	219	0	0	274	546	470	907	0	0	41	100	74	117
Shannon index of diversity*	0.91	0.15	0.99	0.14	0.96	0.14	0.91	0.18	1.13	0.08	0.99	0.11	0.97	0.18	1.03	0.04

* based on feeding groups

Table S2. Mean abundance of enchytraeids (m^{-2}) in April 2013, the Shannon index of diversity and species richness in ambient (A; control), warmed (T), drought (D) and CO_2 treated plots (CO2) at the CLIMAITE field site. Data from Holmstrup et al. (2015).

	A		T		D		TD		CO2		TCO2		DCO2		TDCO2	
	mean	SD	mean	SD	mean	SD	mean	SD	mean	SD	mean	SD	mean	SD	mean	SD
Achaeta aberrans	70	172	0	0	0	0	1053	1298	0	0	0	0	70	172	0	0
Achaeta abulba	0	0	0	0	0	0	0	0	632	1547	351	860	1263	3094	2386	5845
Achaeta affinis	4351	5772	50041	116656	3018	4647	912	2036	983	1426	2035	2546	4492	9777	7439	12747
achaeta sp	140	344	0	0	0	0	1895	4439	0	0	0	0	0	0	1263	3094
Chamaedrilus chlorophilus	20283	17853	30249	21151	21125	10790	24915	26717	18599	14762	25266	20514	24143	11826	18318	12584
Enchytronia parva	281	344	351	414	70	172	491	817	140	217	1263	1975	211	516	1614	2994
Enchytraeus norvegicus	0	0	0	0	0	0	281	510	0	0	140	344	0	0	0	0
Fridericia cylindrica	0	0	0	0	0	0	0	0	0	0	0	0	0	0	281	688
Fridericia striata	0	0	0	0	0	0	0	0	0	0	0	0	0	0	0	0
Marionina clavata	2386	5845	0	0	0	0	70	172	0	0	0	0	0	0	0	0
Oconnorella cambrensis	4281	9674	1333	3266	3299	6248	4492	7494	1404	2317	2597	5764	2246	1740	1263	2498
Fridericia ratzeli	0	0	0	0	0	0	0	0	0	0	0	0	0	0	70	172
Shannon index	0.63818	0.383	0.3343	0.3311	0.4355	0.421	0.6927	0.361	0.3409	0.341	0.463	0.309	0.564	0.21248	0.72316	0.3087
Species richness	3.33	0.82	2.33	1.03	2.33	1.03	3.83	1.60	2.33	1.37	2.83	1.17	3.00	0.63	3.50	1.38

Table S3a. Mean abundance of collembolans (m^{-2}) in October 2007, the Shannon index of diversity and species richness in ambient (A; control), warmed (T), drought (D) and CO_2 treated plots (CO2) at the CLIMAITE field site.

	A		T		D		TD		CO2		TCO2		DCO2		TDCO2	
	mean	SD	mean	SD	mean	SD	mean	SD	mean	SD	mean	SD	mean	SD	mean	SD
Brachystomella parvula	59	144	808	998	883	1994	1242	1437	353	547	375	758	379	758	0	0
Cyphoderus albinus	294	721	0	0	177	432	610	1494	0	0	4321	8911	2936	4409	588	825
Desoria tigrina	0	0	0	0	0	0	0	0	0	0	0	0	0	0	294	721
Entomobrya sp.	118	182	0	0	118	182	0	0	235	365	0	0	379	758	316	774
Folsomia fimetaria	353	865	0	0	0	0	0	0	118	288	0	0	0	0	59	144
Folsomia quadrioculata	883	1667	948	1586	2704	6111	3565	5419	1103	1553	4637	5318	2275	4549	2013	3100
Heteromurus nitidus	0	0	0	0	0	0	353	865	0	0	0	0	0	0	0	0
Isotoma sp.	1536	2232	948	1586	1440	2185	2109	3695	765	1366	632	979	2654	3715	0	0
isotomiella minor	1294	2685	59	144	59	144	5798	8583	412	1009	1954	3759	424	684	14886	35436
Isotomurus sp.	0	0	0	0	632	1548	316	774	0	0	0	0	0	0	0	0
Lepidocyrtus lanuginosus	59	144	0	0	0	0	0	0	0	0	0	0	0	0	316	774
Megalothorax minimus	10546	19161	5110	5221	5079	4288	4506	6844	2706	3632	4460	3499	1252	882	5166	3739
Mesaphorura sp.	6376	4958	21037	28053	17554	15835	5175	6410	18527	32137	9143	6815	11403	14155	13352	8864
Micranurida pygmaea	118	288	669	1032	867	1615	706	1023	412	266	1065	1693	1896	2937	551	744
Micranurophorus musci	1059	2594	471	763	1589	2484	412	1009	1942	4756	3707	9079	0	0	1589	3891
Neanura muscorum	316	774	316	774	59	144	59	144	235	428	0	0	141	173	0	0
Orchesella sp.	375	758	316	774	316	774	0	0	118	288	0	0	0	0	316	774
Parisotoma notabilis	6655	5101	2756	1770	4578	4832	8696	7734	4896	4417	12674	14387	10087	15029	13080	16064
Protaphorura sp.	2360	2812	3432	2460	5783	6721	9862	15046	2279	1313	7325	7064	7274	6478	4953	5511
Sminthuridae spp.	1669	664	59	144	1440	2185	728	898	706	1023	177	295	2107	2242	2013	1540
Sminthurides pumilis	412	469	59	144	59	144	59	144	118	182	0	0	0	0	316	774
Tomocerus sp.	375	758	0	0	0	0	59	144	0	0	0	0	0	0	375	758
Willemia sp.	0	0	0	0	316	774	0	0	0	0	0	0	0	0	0	0
Shannon index	1.64	0.31	1.28	0.44	1.47	0.42	1.70	0.26	1.40	0.55	1.48	0.42	1.53	0.41	1.53	0.26
Species richness	8.50	2.81	6.67	1.51	8.33	1.63	8.17	2.32	7.83	3.66	6.67	1.51	7.50	3.51	8.17	1.72

Table S3b. Mean abundance of collembolans (m^{-2}) in April 2013, the Shannon index of diversity and species richness in ambient (A; control), warmed (T), drought (D) and CO_2 treated plots (CO2) at the CLIMAITE field site.

	A		T		D		TD		CO2		TCO2		DCO2		TDCO2	
	mean	SD	mean	SD	mean	SD	mean	SD	mean	SD	mean	SD	mean	SD	mean	SD
Brachystomelle parvula	633	980	316	775	316	775	1266	1961	316	775	949	2325	949	1040	949	1040
Cyphoderus albinus	118	289	0	0	316	775	59	144	0	0	670	1040	412	649	0	0
Entomobryidae spp	3164	6082	633	980	434	738	1898	1201	1957	3137	1898	3797	1582	2219	692	1528
Folsomia manolachei	5379	9018	1008	1716	2847	2325	3480	5425	3164	2299	1898	3797	2847	1991	1266	1550
Isotoma viridis	0	0	316	775	0	0	0	0	0	0	0	0	0	0	0	0
Isotomiella minor	33338	33232	20248	19122	19535	19730	20168	19184	41542	52650	115259	69396	53072	104872	31499	21384
Lepidocyrtus violaceus	0	0	0	0	0	0	0	0	0	0	0	0	0	0	0	0
Lepidocyrtus_lignorum	0	0	316	775	316	775	633	1550	633	980	633	980	1067	1681	59	144
Megalothorax minimus	8440	6830	3856	3678	5872	7214	10500	14804	4172	4364	6799	3035	10500	12711	4606	3322
Micranurida pygmaea	3539	2234	692	1528	1266	980	633	980	1501	655	1759	1537	1582	1429	4113	4398
Micranurophorus musci	0	0	1237	3031	4772	11690	589	1443	0	0	295	722	177	296	766	1876
Neanura muscorum	0	0	0	0	0	0	0	0	316	775	0	0	0	0	0	0
Onychiurinae spp	2531	2299	1898	2941	4113	6400	4746	4611	1898	2401	633	980	5695	5233	4805	8862
Orchesella sp	0	0	316	775	316	775	316	775	0	0	316	775	0	0	316	775
Parisotoma notabilis	11390	16462	14870	19997	8226	9117	8918	9073	16768	16480	15186	11640	11625	7842	8542	4914
Pogonognathellus flavesens	1266	3100	633	980	1582	2523	2847	4287	0	0	633	980	949	1588	316	775
Sminthurides pumilis	0	0	0	0	0	0	0	0	0	0	316	775	316	775	0	0
Symphypleona spp	2016	1708	3458	4537	8344	6638	7395	4381	3856	2787	7137	5075	6821	7298	4408	5386
Tullbergiinae spp	26026	10220	9330	8287	51381	52403	48996	58247	11169	8294	17270	8689	19454	16778	15003	11818
Willemia sp	0	0	1898	4650	0	0	0	0	0	0	0	0	0	0	0	0
Shannon index	1.46	0.30	1.61	0.37	1.38	0.32	1.43	0.59	1.49	0.35	1.22	0.40	1.68	0.38	1.49	0.17
Species richness	7.67	1.03	7.50	1.87	8.67	1.86	8.00	1.67	8.00	1.79	8.33	2.07	9.33	1.75	7.67	1.75

Table S4a. Mean abundance of oribatids (m^{-2}) in 2007, the Shannon index of diversity and species richness in ambient (A; control), warmed (T), drought (D) and CO_2 treated plots (CO2) at the CLIMAITE field site.

	A		T		D		TD		CO2		TCO2		DCO2		TDCO2	
	mean	SD	mean	SD	mean	SD	mean	SD	mean	SD	mean	SD	mean	SD	mean	SD
Achipteria coleoptrata	0	0	0	0	0	0	118	289	0	0	0	0	0	0	0	0
Belba sp	412	849	236	428	354	447	471	695	177	433	530	698	0	0	177	296
Brachychochthonius zelawaiensis	0	0	177	296	0	0	530	829	648	1010	118	289	0	0	884	1840
Camisia spinifer	177	296	0	0	177	433	0	0	59	144	0	0	59	144	118	183
Carabodes forsslundi	0	0	0	0	177	433	5008	12268	1120	2742	0	0	0	0	59	144
Eupelops acromios	118	183	0	0	0	0	412	649	0	0	118	289	59	144	0	0
Galumna lanceata	884	1410	118	183	589	940	177	433	471	483	589	730	59	144	1591	2426
Galumna obvia	354	866	1120	1523	884	1479	648	756	2416	2867	1355	1808	707	671	295	348
Hemileius initialis	59	144	0	0	0	0	0	0	59	144	0	0	0	0	0	0
Heminothrus peltifer	236	577	59	144	0	0	2062	5052	0	0	236	428	236	365	2357	4958
Latilamellobates incisellus	0	0	0	0	59	144	59	144	0	0	0	0	0	0	0	0
Liacarus coracinus	177	433	118	183	295	470	59	144	118	183	0	0	0	0	118	183
Liochthonius sp	0	0	0	0	0	0	0	0	0	0	0	0	0	0	295	722
Malaconothrus monodactylus	59	144	59	144	0	0	0	0	0	0	0	0	0	0	0	0
Metabelba sp	0	0	0	0	118	289	0	0	0	0	59	144	236	577	0	0
Microppia minus	1650	4041	4478	5931	707	1565	2475	2932	2357	3096	1178	2141	0	0	3830	3971
Moritzoppia unicarinata	0	0	0	0	118	289	0	0	0	0	295	722	0	0	530	887
Nanhermannia comitalis	0	0	0	0	0	0	0	0	0	0	0	0	0	0	118	289
Nanhermannia nanus	59	144	0	0	0	0	0	0	0	0	0	0	0	0	0	0
Nothrus anauniensis	1473	1738	2652	3638	2887	2592	1414	2037	5421	11401	2887	4485	0	0	1061	1949
Nothrus silvestris	0	0	118	289	0	0	707	806	589	1443	0	0	0	0	884	2165
Oppiella nova	0	0	118	289	177	433	0	0	0	0	295	722	295	566	0	0
Oribatula tibialis	59	144	0	0	0	0	0	0	0	0	0	0	0	0	0	0
Paradamaeus clavipes	59	144	0	0	0	0	0	0	0	0	0	0	0	0	0	0
Peloptulus reticulatus	0	0	0	0	0	0	118	289	0	0	0	0	236	577	0	0
Phthiracarus laevigatus	59	144	59	144	0	0	0	0	0	0	0	0	59	144	0	0
Rhysotritia duplicata	0	0	0	0	177	296	0	0	0	0	0	0	0	0	0	0
Scheloribates laevigatus	1002	1035	825	1443	1120	1059	1532	2415	3653	2526	3476	5032	2534	3552	3653	913
Scutovertex minutus	59	144	0	0	648	1588	59	144	0	0	0	0	0	0	0	0
Suctobelbella acutidens	707	975	1178	2141	1414	1565	1061	1414	1886	2546	118	289	0	0	1061	1658
Suctobelbella alloenasuta	648	566	884	1529	471	619	2062	2919	177	433	589	966	0	0	1532	1278
Suctobelbella sarekensis	0	0	177	433	0	0	0	0	118	289	118	289	59	144	295	470
Suctobelbella subtrigona	118	289	177	433	295	722	471	827	589	1133	0	0	0	0	589	913
Tectocepheus sarekensis	0	0	1944	3254	236	577	1237	1512	412	1010	0	0	1237	1946	118	289
Tectocepheus velatus	1650	3706	7542	8073	8308	10706	2298	1984	2062	3158	2652	2835	0	0	2946	4293
Shannon index	1.46	0.49	1.45	0.25	1.38	0.50	1.64	0.49	1.42	0.58	1.38	0.44	0.98	0.49	1.69	0.41
Species richness	6.50	2.74	6.67	1.63	7.33	2.73	8.50	2.66	6.83	3.71	6.00	2.83	3.50	1.64	8.50	2.59

Table S4b. Mean abundance of oribatids (m^{-2}) in 2013, the Shannon index of diversity and species richness in ambient (A; control), warmed (T), drought (D) and CO_2 treated plots (CO2) at the CLIMAITE field site.

	A		T		D		TD		CO2		TCO2		DCO2		TDCO2	
	mean	SD	mean	SD	mean	SD	mean	SD	mean	SD	mean	SD	mean	SD	mean	SD
Berniniella sigma	423	373	0	0	0	0	2459	4259	0	0	5568	9645	0	0	0	0
Brachychthoniidae sp	1815	3143	0	0	594	1029	5169	6399	2859	4951	0	0	16667	28868	0	0
Brachychthonius bimaculatus	0	0	0	0	0	0	316	547	0	0	0	0	0	0	0	0
Brachychthonius hirtus	563	976	0	0	0	0	0	0	0	0	0	0	0	0	0	0
Brachychthonius impressus	3670	3208	9329	12564	434	380	948	1642	0	0	236	408	0	0	471	540
Brachychthonius sp	165	286	0	0	396	686	1612	2792	0	0	3969	6570	0	0	0	0
Carabodes marginatus	0	0	0	0	0	0	2710	2654	1178	1472	0	0	0	0	0	0
Cepheus cepheiformes	0	0	0	0	118	204	0	0	0	0	0	0	0	0	0	0
Chamobates pusillus	2003	1947	943	1633	1532	2654	354	612	0	0	0	0	118	204	0	0
Cultroribula bicultrata	118	204	1532	2354	0	0	0	0	0	0	0	0	0	0	0	0
Damaeidae sp	118	204	0	0	0	0	118	204	0	0	0	0	0	0	0	0
Dissorhina ornata	1178	2041	2946	3007	1943	2254	0	0	3566	6177	3550	3535	1625	2814	825	1429
Eupelops acromius	0	0	0	0	0	0	0	0	118	204	0	0	0	0	0	0
Eupelops occultus	0	0	354	354	0	0	0	0	0	0	0	0	0	0	0	0
Eupelops plicatus	118	204	1061	707	0	0	0	0	1061	1837	0	0	471	540	0	0
Galumna lanceata	589	736	0	0	0	0	0	0	118	204	0	0	236	204	0	0
Galumna obvia	354	354	118	204	1061	1837	0	0	118	204	1886	1472	2828	2669	236	408
Damaeidae juv	1328	1247	825	736	1296	1136	0	0	1532	2654	0	0	0	0	0	0
Nothridae unidentified juveniles	15485	9166	6364	7483	4007	6636	0	0	72483	53477	0	0	0	0	5067	8777
Oribatida unidentified juveniles	30939	45638	2357	4082	14467	20798	33095	21515	589	540	30987	18596	57463	15734	23569	33376
Liacarus coracinus	825	1136	118	204	236	204	118	204	354	612	236	408	471	204	236	204
Liebstadia similis	354	354	3535	3535	1532	2354	118	204	825	540	2121	3062	589	540	236	408
Liochthonius alpestris	0	0	0	0	198	343	322	558	0	0	0	0	0	0	0	0
Liochthonius brevis	3380	5854	0	0	4751	8228	5047	5949	715	1238	13611	952	11584	7904	3771	3559
Liochthonius dilutus	0	0	1250	2165	198	343	322	558	357	619	0	0	0	0	0	0
Liochthonius evansi	282	488	0	0	0	0	0	0	0	0	241	417	383	663	236	408
Liochthonius furcillatus	0	0	0	0	0	0	0	0	0	0	118	204	0	0	0	0
Liochthonius hystericinus	330	572	5632	6923	198	343	0	0	0	0	0	0	464	804	0	0
Liochthonius lapponicus	0	0	0	0	0	0	471	816	1072	1857	0	0	0	0	0	0
Liochthonius leptalus	0	0	0	0	0	0	354	612	0	0	2169	3757	1857	3216	0	0
Liochthonius muscorum	282	488	0	0	0	0	0	0	0	0	2605	2275	427	740	0	0
Liochthonius neglectus	495	857	2475	4286	792	1371	0	0	0	0	0	0	0	0	0	0
Liochthonius perfosorius	0	0	0	0	0	0	0	0	1208	1795	589	1021	0	0	471	816
Liochthonius propinquus	0	0	0	0	354	612	0	0	0	0	0	0	0	0	0	0
Liochthonius simplex	0	0	0	0	990	1714	118	204	0	0	0	0	0	0	0	0
Liochthonius sp	4000	3586	589	1021	2771	4800	2114	1959	707	1225	1645	1315	5791	2633	1296	540
Liochthonius tuxeni	563	976	0	0	0	0	0	0	0	0	0	0	0	0	118	204

Table S4b continued

Metabelba pulverolenta	354	354	589	736	1650	2010	0	0	2475	4286	825	1136	707	354	0	0
Microppia minus	4606	7674	7660	11770	2167	2154	10905	12999	20859	30620	23670	32183	13036	17292	4714	5295
Neobrachychthonius sp	330	572	0	0	0	0	1896	3284	0	0	0	0	0	0	0	0
Neoliochthonius globuliferus	282	488	0	0	792	1371	2357	4082	357	619	350	606	855	1480	236	408
Neoliochthonius sp	0	0	0	0	0	0	434	479	0	0	0	0	0	0	0	0
Nothrus anauensis	118	204	0	0	0	0	0	0	354	612	0	0	0	0	0	0
Nothrus silvestris	3418	2131	3064	5004	1532	2041	2121	1968	1650	1472	3535	3984	4125	5412	2357	2483
Oppeiidae sp	0	0	0	0	118	204	0	0	0	0	0	0	0	0	0	0
Oppiella fallax	0	0	0	0	817	1416	0	0	0	0	0	0	0	0	0	0
Oppiella nova	0	0	0	0	163	283	3399	4446	3182	5208	2475	2894	4418	1549	118	204
Oppiella sp	236	408	0	0	490	849	0	0	0	0	0	0	244	423	0	0
Oribatula tibialis	943	890	0	0	0	0	0	0	354	354	354	0	354	354	943	1338
Peloptulus phaenotus	0	0	0	0	354	354	471	816	1178	2041	0	0	118	204	118	204
Pergalumna nervosa	1178	2041	589	540	1296	408	1178	408	2475	2151	1061	1837	471	408	354	612
Platynothrus peltifer	118	204	2121	2806	2357	2131	0	0	118	204	236	204	4950	8573	0	0
Porobelba spinosa	236	408	0	0	0	0	0	0	0	0	0	0	0	0	0	0
Quadroppia sp	728	631	0	0	1061	1837	354	612	118	204	250	218	0	0	236	408
Scutovertex minutus	0	0	0	0	0	0	825	1429	0	0	0	0	0	0	0	0
Scheloribates laevigatus	0	0	236	204	825	890	118	204	1296	408	2710	2700	2710	1242	2003	2131
Scheloribates latipes	0	0	0	0	0	0	0	0	0	0	0	0	236	204	0	0
Scheloribates sp	0	0	0	0	118	204	0	0	0	0	354	612	0	0	0	0
Sellnickochthonius cricoides	2302	3168	0	0	396	686	1264	2190	0	0	2368	2939	1764	2477	236	408
Sellnickochthonius furcatus	0	0	0	0	0	0	8187	10975	2501	4333	0	0	998	1178	354	354
Sellnickochthonius hungaricus	0	0	0	0	0	0	0	0	0	0	241	417	0	0	0	0
Sellnickochthonius immaculatus	1247	385	0	0	396	686	1896	3284	12305	21313	0	0	810	705	471	816
Sellnickochthonius jacoti	0	0	0	0	0	0	4507	7001	22513	38993	5144	6613	11344	19048	707	1225
Sellnickochthonius suecicus	0	0	0	0	8710	15085	7159	5479	1072	1857	11127	14012	29913	18004	3064	5307
Sellnickochthonius sp	282	488	0	0	792	1371	10450	9097	1787	3095	9532	6212	20692	15061	1061	1275
Sellnickochthonius zelawaiensis	30092	24988	4928	5752	2253	2769	8289	12924	4517	7824	19584	14437	37842	7971	2121	2761
Suctobelbella acutidens	18088	19617	3510	4126	3102	1070	6685	7473	4832	4392	4434	2118	8616	3082	1532	1594
Suctobelbella diffusa	0	0	0	0	0	0	0	0	0	0	589	1021	232	402	0	0
Suctobelbella falcata	0	0	1403	2430	1532	2654	236	408	2593	3894	377	653	1649	1748	236	408
Suctobelbella prominens	187	324	342	593	0	0	0	0	0	0	0	0	0	0	118	204
Suctobelbella sarakensis	423	373	3384	3545	236	408	2810	4867	2475	2894	3753	3241	2807	2600	589	540
Suctobelbella similis	561	972	0	0	163	283	1054	1825	471	816	1559	862	2100	740	354	354
Suctobelbella sp	3395	4437	513	889	589	1021	943	1633	1414	2449	2042	1797	3675	4760	1061	935
Suctobelbella subcornigera	3707	3268	2632	3962	1931	2022	7968	11439	354	612	5162	5092	12107	13371	5421	4748
Suctobelbella subtrigona	7515	5262	0	0	3011	3309	703	1217	11400	16455	2640	4572	1610	1411	825	890
Suctobelbella vera	11021	15841	825	1429	1414	1620	4462	3924	236	408	3208	1138	7195	8379	4007	3470
Tectocepheus velatus alatus	0	0	0	0	236	204	236	408	589	1021	118	204	118	204	0	0
Tectocepheus velatus sarakensis	1296	1136	825	1429	3182	2208	4832	4614	1414	1620	2003	1594	589	408	589	1021
Tectocepheus velatus spp	471	816	118	204	0	0	0	0	12963	22453	0	0	0	0	236	408
Tectocepheus velatus velatus	118	204	0	0	825	736	3535	5820	0	0	1296	1021	707	707	707	935
Trichoribates incisellus	236	204	0	0	707	1225	118	204	707	1225	0	0	0	0	0	0
Verachthonius congruus	330	572	118	204	0	0	0	0	0	0	0	0	0	0	0	0
Shannon index	2.38	0.73	2.37	0.17	2.75	0.16	2.61	0.10	2.04	0.25	2.66	0.27	2.54	0.03	2.29	0.32
Species richness	29.00	13.00	18.33	7.37	27.33	9.71	24.67	7.02	23.00	7.00	30.67	2.52	31.67	2.31	21.33	9.61

Table S5a. Summary statistics of the Structural Equation Modelling of 2007 data. Exogenous factors are shown with capitals. Significant correlations are shown in **bold**.

**Parameter**	Estimate	Standard Error	Z-value	P(>|z|)	Standardized regression coefficients
Soil water content during drought					
DROUGHT	-0.91	0.046	-19.872	**<0.001**	**-0.944**
Soil water content (mean annual)					
WARMING	-1.311	0.373	-3.515	**<0.001**	**-0.419**
Soil water content during drought	1.221	0.39	3.132	**0.002**	**0.376**
CO_2	-0.179	0.375	-0.476	0.634	-0.057
Soil temperature 5 cm (mean annual)					
WARMING	0.203	0.031	6.597	**<0.001**	**0.657**
CO_2	0.094	0.031	3.056	**0.002**	**0.304**
Litter C:N					
Soil water content during drought	0.01	0.376	0.026	0.979	0.004
Soil water content (mean annual)	0.183	0.115	1.595	0.111	0.224
Soil temperature 5 cm (mean annual)	-1.81	1.143	-1.583	0.113	-0.218
CO_2	1.089	0.343	3.177	**0.001**	**0.425**
Fungi:bacteria ratio					
Soil water content during drought	0.016	0.027	0.597	0.551	0.088
Soil water content (mean annual)	-0.012	0.008	-1.48	0.139	-0.222
Soil temperature 5 cm (mean annual)	0.192	0.083	2.302	**0.021**	**0.339**
CO_2	-0.046	0.027	-1.71	0.087	-0.262
Litter C:N	0.017	0.01	1.619	0.105	0.244
Microbial C:N					
Soil water content during drought	-1.184	1.055	-1.122	0.262	-0.177
Soil water content (mean annual)	0.208	0.326	0.638	0.524	0.101
Soil temperature 5 cm (mean annual)	2.031	3.322	0.611	0.541	0.097
CO_2	-0.573	0.972	-0.59	0.555	-0.089
Fungi:bacteria ratio	-6.414	5.538	-1.158	0.247	-0.173
Collembola diversity (Shannon)					
Soil water content during drought	-0.13	0.122	-1.066	0.287	-0.161
Soil water content (mean annual)	0.007	0.038	0.17	0.865	0.026
Soil temperature 5 cm (mean annual)	0.56	0.402	1.392	0.164	0.222
CO_2	-0.214	0.128	-1.675	0.094	-0.275
Litter C:N	0.109	0.051	2.143	**0.032**	**0.358**
Fungi:bacteria ratio	-1.116	0.67	-1.665	0.096	-0.251
Microbial C:N	-0.018	0.018	-1.003	0.316	-0.147

Table S5a continued

Oribatida diversity (Shannon)					
Soil water content during drought	-0.004	0.137	-0.03	0.976	-0.004
Soil water content (mean annual)	0.041	0.043	0.94	0.347	0.133
Soil temperature 5 cm (mean annual)	0.299	0.452	0.662	0.508	0.096
CO_2	0.048	0.144	0.334	0.739	0.05
Litter C:N	-0.204	0.057	-3.571	**<0.001**	**-0.545**
Fungi:bacteria ratio	0.066	0.754	0.087	0.93	0.012
Microbial C:N	0.018	0.02	0.929	0.353	0.124
Nematode functional diversity (Shannon)					
Soil water content during drought	-0.012	0.057	-0.208	0.835	-0.027
Soil water content (mean annual)	-0.028	0.018	-1.566	0.117	-0.207
Soil temperature 5 cm (mean annual)	-0.058	0.189	-0.308	0.758	-0.043
CO_2	0.022	0.06	0.361	0.718	0.051
Litter C:N	0.075	0.023	3.209	**0.001**	**0.458**
Fungi:bacteria ratio	-0.136	0.311	-0.438	0.661	-0.057
Microbial C:N	-0.025	0.008	-3.057	**0.002**	**-0.388**
**Covariances**					
Collembola diversity (Shannon)					
Oribatida diversity (Shannon)	0.019	0.021	0.932	0.351	0.136
Nematode functional diversity (Shannon)	0.016	0.009	1.769	0.077	0.27
Oribatida diversity (Shannon)					
Nematode functional diversity (Shannon)	0.009	0.01	0.909	0.363	0.133
**Variances**					
	Estimate	Standard Error			R^2
Soil water content during drought	0.025	0.005			0.892
Soil water content (mean annual)	1.666	0.34			0.32
Soil temperature 5 cm (mean annual)	0.011	0.002			0.524
Litter C:N	1.248	0.255			0.24
Fungi:bacteria ratio	0.006	0.001			0.174
Microbial C:N	9.777	1.996			0.063
Collembola diversity (Shannon)	0.134	0.027			0.161
Oribatida diversity (Shannon)	0.161	0.033			0.301
Nematode functional diversity (Shannon)	0.026	0.005			0.389

Table S5b. Summary statistics of the Structural Equation Modelling of 2013 data. Exogenous factors are shown with capitals. Significant correlations are shown in **bold**.

Parameter	Estimate	Standard Error	Z-value	P(>|z|)	Standardized regression coefficients
Soil water content during drought					
DROUGHT	-0.611	0.068	-9.049	**<0.001**	**-0.794**
Soil water content (mean annual)					
WARMING	-0.964	0.474	-2.035	**0.042**	**-0.233**
Soil water content during drought	3.023	0.616	4.91	**<0.001**	**0.562**
CO_2	-0.124	0.474	-0.262	0.793	-0.03
Soil temperature 5 cm (mean annual)					
WARMING	0.067	0.038	1.747	0.081	0.236
CO_2	0.076	0.038	1.978	**0.048**	**0.267**
Litter C:N					
Soil water content during drought	0.095	0.132	0.72	0.472	0.115
Soil water content (mean annual)	-0.026	0.024	-1.078	0.281	-0.171
Soil temperature 5 cm (mean annual)	-0.16	0.313	-0.51	0.61	-0.071
CO_2	0.264	0.087	3.054	**0.002**	**0.415**
Fungi:bacteria ratio					
Soil water content during drought	0.017	0.086	0.2	0.841	0.034
Soil water content (mean annual)	-0.013	0.016	-0.79	0.43	-0.136
Soil temperature 5 cm (mean annual)	0.035	0.204	0.173	0.862	0.026
CO_2	-0.046	0.061	-0.742	0.458	-0.118
Litter C:N	0.133	0.094	1.425	0.154	0.221
Microbial C:N					
Soil water content during drought	0.697	0.798	0.874	0.382	0.151
Soil water content (mean annual)	-0.063	0.149	-0.422	0.673	-0.073
Soil temperature 5 cm (mean annual)	1.138	1.906	0.597	0.55	0.091
CO_2	0.044	0.524	0.084	0.933	0.012
Fungi:bacteria ratio	1.375	1.317	1.044	0.297	0.149
Enchytraeidae diversity (Shannon)					
Soil water content during drought	-0.111	0.068	-1.641	0.101	-0.277
Soil water content (mean annual)	0.021	0.013	1.636	0.102	0.276
Soil temperature 5 cm (mean annual)	0.106	0.16	0.663	0.507	0.097
CO_2	0.003	0.048	0.069	0.945	0.011
Litter C:N	-0.014	0.075	-0.184	0.854	-0.028
Fungi:bacteria ratio	0.028	0.114	0.247	0.805	0.035
Microbial C:N	-0.016	0.012	-1.289	0.197	-0.181
Collembola diversity (Shannon)					
Soil water content during drought	-0.211	0.156	-1.352	0.176	-0.222
Soil water content (mean annual)	0.079	0.029	2.718	**0.007**	**0.446**
Soil temperature 5 cm (mean annual)	-0.106	0.368	-0.287	0.774	-0.041
CO_2	0.053	0.111	0.479	0.632	0.073
Litter C:N	-0.146	0.172	-0.845	0.398	-0.126
Fungi:bacteria ratio	0.123	0.263	0.468	0.64	0.065
Microbial C:N	0.017	0.028	0.593	0.553	0.081

Table S5b continued

Oribatida diversity (Shannon)					
Soil water content during drought	-0.022	0.201	-0.111	0.912	-0.023
Soil water content (mean annual)	0.03	0.036	0.847	0.397	0.167
Soil temperature 5 cm (mean annual)	-0.145	0.536	-0.27	0.787	-0.055
CO_2	-0.311	0.16	-1.949	0.051	-0.418
Litter C:N	0.524	0.31	1.691	0.091	0.448
Fungi:bacteria ratio	0.277	0.381	0.727	0.467	0.143
Microbial C:N	0.025	0.06	0.414	0.679	0.119
Nematode functional diversity (Shannon)					
Soil water content during drought	0.067	0.056	1.198	0.231	0.187
Soil water content (mean annual)	-0.018	0.01	-1.705	0.088	-0.266
Soil temperature 5 cm (mean annual)	-0.166	0.132	-1.258	0.208	-0.17
CO_2	0.108	0.04	2.699	**0.007**	**0.389**
Litter C:N	-0.036	0.062	-0.585	0.559	-0.083
Fungi:bacteria ratio	0.075	0.094	0.797	0.425	0.105
Microbial C:N	0.017	0.01	1.634	0.102	0.213
**Covariances**					
Enchytraeidae diversity (Shannon)					
Collembola diversity (Shannon)	-0.006	0.007	-0.798	0.425	-0.116
Oribatida diversity (Shannon)	0.008	0.01	0.822	0.411	0.174
Nematode functional diversity (Shannon)	0.002	0.003	0.832	0.405	0.121
Collembola diversity (Shannon)					
Oribatida diversity (Shannon)	0.018	0.029	0.642	0.521	0.176
Nematode functional diversity (Shannon)	0.001	0.006	0.098	0.922	0.014
Oribatida diversity (Shannon)					
Nematode functional diversity (Shannon)	-0.003	0.01	-0.333	0.739	-0.09
**Variances**					
	Estimate	Standard Error			R^2
Soil water content during drought	0.055	0.011			0.63
Soil water content (mean annual)	2.694	0.55			0.371
Soil temperature 5 cm (mean annual)	0.018	0.004			0.127
Litter C:N	0.083	0.017			0.185
Fungi:bacteria ratio	0.035	0.007			0.062
Microbial C:N	3.003	0.62			0.049
Enchytraeidae diversity (Shannon)	0.021	0.004			0.114
Collembola diversity (Shannon)	0.113	0.023			0.161
Oribatida diversity (Shannon)	0.096	0.028			0.309
Nematode functional diversity (Shannon)	0.015	0.003			0.243

Table S6. Mean soil temperature during 12 months prior sampling (Ts5; 5 cm depth), mean soil water content during 12 months prior sampling (SWC; 0-20 cm depth) and minimum SWC during drought (0-20 cm depth) in ambient (A; control), warmed (T), drought (D) and CO_2 treated plots (CO2) at the CLIMAITE field site.

	A		T		D		TD		CO2		TCO2		DCO2		TDCO2	
	mean	SD	mean	SD	mean	SD	mean	SD	mean	SD	mean	SD	mean	SD	mean	SD
2007																
mean Ts5 12 months prior sampling (°C)	10.29	0.12	10.55	0.12	10.27	0.06	10.42	0.13	10.37	0.12	10.59	0.10	10.38	0.12	10.56	0.11
mean SWC 12 months prior sampling (vol%)	17.74	0.68	16.00	1.84	15.58	0.97	14.88	2.05	16.18	1.42	15.61	1.11	16.79	1.30	14.36	0.76
Minimum SWC during drought (vol%)*	16.51	0.66	14.75	1.90	5.51	0.62	6.18	2.91	13.89	2.13	13.73	1.11	5.28	0.68	4.70	0.78
2013																
mean Ts5 12 months prior sampling (°C)	8.05	0.18	8.17	0.14	8.02	0.12	8.07	0.16	8.12	0.10	8.20	0.14	8.14	0.14	8.17	0.13
mean SWC 12 months prior sampling (vol%)	17.11	0.47	14.98	2.33	15.28	0.77	14.35	3.43	14.78	1.87	15.79	1.57	16.25	1.57	14.21	2.60
Minimum SWC during drought (vol%)**	16.28	1.66	13.78	3.69	7.26	0.99	8.17	1.62	13.72	3.40	16.08	1.86	8.14	1.24	7.60	4.89

* the mean SWC during the last week (June 15-22, 2007) of the latest drought period (May 21 to June 22, 2007)

** the mean SWC during the last week (4-11 June 2012) of the latest drought period (8 May to 11 June)

Table S7. Mean litter C:N, Fungi:bacteria ratio and microbial C:N in ambient (A; control), warmed (T), drought (D) and CO_2 treated plots (CO2) at the CLIMAITE field site. Litter C:N (2013) and microbial C:N (2013) are from Vestergård et al. (2015).

	A		T		D		TD		CO2		TCO2		DCO2		TDCO2	
	mean	SD	mean	SD	mean	SD	mean	SD	mean	SD	mean	SD	mean	SD	mean	SD
2007																
Litter C:N ratio	41.62	6.08	40.38	10.17	38.95	3.35	38.00	5.47	49.26	11.15	45.57	13.20	61.13	21.69	37.38	7.79
Fungi:bacteria ratio	0.10	0.09	0.19	0.09	0.14	0.09	0.23	0.12	0.12	0.11	0.24	0.07	0.17	0.12	0.09	0.07
Microbial C:N ratio	9.62	2.93	8.87	2.15	8.99	2.70	10.00	3.17	9.65	5.39	7.29	3.23	10.16	3.88	9.41	2.79
2013																
Litter C:N ratio	26.75	3.66	26.90	3.94	27.92	2.53	28.43	2.59	31.74	3.11	29.37	2.16	29.37	2.37	29.73	3.60
Fungi:bacteria ratio	0.66	0.13	0.65	0.21	0.67	0.25	0.60	0.11	0.75	0.13	0.54	0.14	0.67	0.34	0.60	0.18
Microbial C:N ratio	8.57	0.58	9.82	1.45	9.92	1.77	9.70	0.96	10.80	3.45	9.70	1.97	8.65	1.00	9.13	1.55

Figure S1. Additional degree days (i.e. accumulated hourly residuals) in elevated temperature plots compared to non-elevated temperature plots (*N* = 24) during the period 2006-2013. Graphs are zeroed 1st of January each year. Grey areas represent 1 standard error.

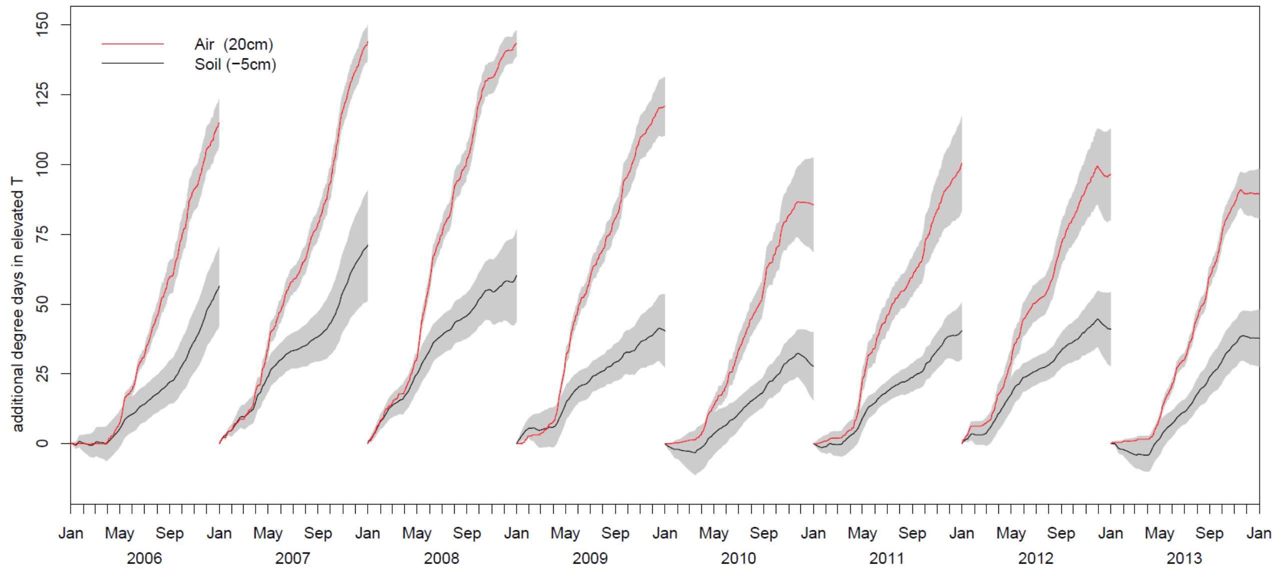

References

Holmstrup, M., Schmelz, R.M., Carrera, N., Dyrnum, K., Larsen, K.S., Mikkelsen, T.N., Beier, C., 2015. Responses of enchytraeids to increased temperature, drought and atmospheric CO_2: Results of an eight-year field experiment in dry heathland. European Journal of Soil Biology 70, 15-22.

Vestergård, M., Dyrnum, K., Michelsen, A., Damgaard, C., Holmstrup, M., 2015. Long-term multifactorial climate change impacts on mesofaunal biomass and nitrogen content. Applied Soil Ecology 92, 54-63.
